# Gastroesophageal Cancer After Laparoscopic Sleeve Gastrectomy

**DOI:** 10.7759/cureus.53435

**Published:** 2024-02-02

**Authors:** Mansour Alkhurmudi, Abdullah S Alzaharani, Talal A Almutairi

**Affiliations:** 1 General Surgery, Prince Sultan Military Medical City, Riyadh, SAU

**Keywords:** gerd, esophageal cancer, gastric cancer, sleeve gastrectomy, cancer, obesity

## Abstract

Obesity has been linked to many types of cancers, and this association has received much attention. Here, we are reporting the case of a 41-year-old male patient, the second case diagnosed in our hospital with advanced metastatic gastroesophageal cancer eight years after laparoscopic sleeve gastrectomy.

Routine preoperative endoscopy for all patients planned for bariatric surgery can play an important role in preoperative surgery selection, detection of abnormal pathology/lesions, as well as in postoperative follow-up/esophagogastroduodenoscopy surveillance plans, especially for patients identified as high-risk to develop cancer.

## Introduction

The prevalence of obesity is increasing worldwide [1], resulting in different surgical and non-surgical approaches to manage this disease and its comorbidities. Obesity increases the risk of gastroesophageal reflux disease (GERD) which can progress into Barrett’s esophagus, which may indirectly increase the risk of esophageal adenocarcinoma [2].

This case report aims to highlight the importance of preoperative esophagogastroduodenoscopy (EGD) in all patients planned for bariatric surgery even in the absence of GERD symptoms. In addition, postoperative surveillance EGD can aid in the early diagnosis of gastroesophageal cancer, especially in high-risk patients.

## Case presentation

The patient was a 41-year-old male, active smoker, with no significant prior medical history, and surgical history notable for laparoscopic sleeve gastrectomy performed at an outside hospital eight years prior. His body mass index (BMI) before the bariatric surgery was 53 kg/m^2^. He was previously healthy until he presented to his primary care physician with complaints of generalized fatigue and decreased appetite. There was no history of GERD or dysphagia symptoms.

Basic investigations at that time showed initially low hemoglobin level (5.9 g/L), for which he received two units of packed red blood cells. All tumor markers were within normal limits. Further investigations and imaging showed a lower esophageal/upper stomach mass, for which he was referred to an upper gastrointestinal surgery clinic.

**Figure 1:**
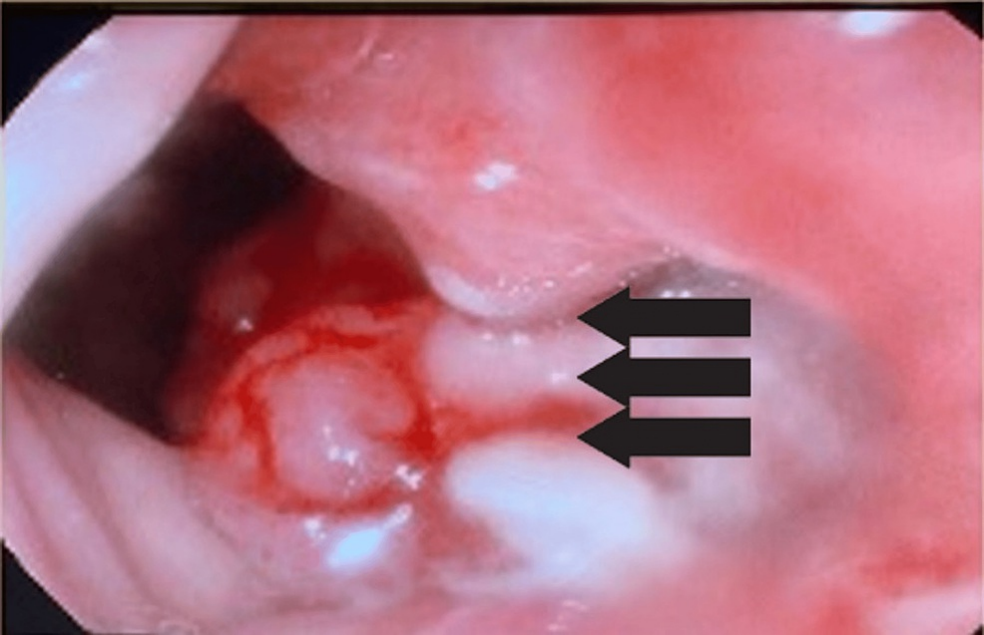
Circumferential fungating mass at the lower esophagus.

The patient was admitted for further investigations, which revealed a lower esophageal mass extending to the proximal stomach as well as the lower esophagus on CT of the abdomen. Furthermore, his CT chest showed left lower cervical, left upper paratracheal, and right lower paravertebral enlarged lymph nodes. He also underwent EGD which showed a lower esophagus circumferential fungating mass extending to the cardia toward lesser curvature, for which multiple biopsies were taken (Figure 1).

**Figure 2:**
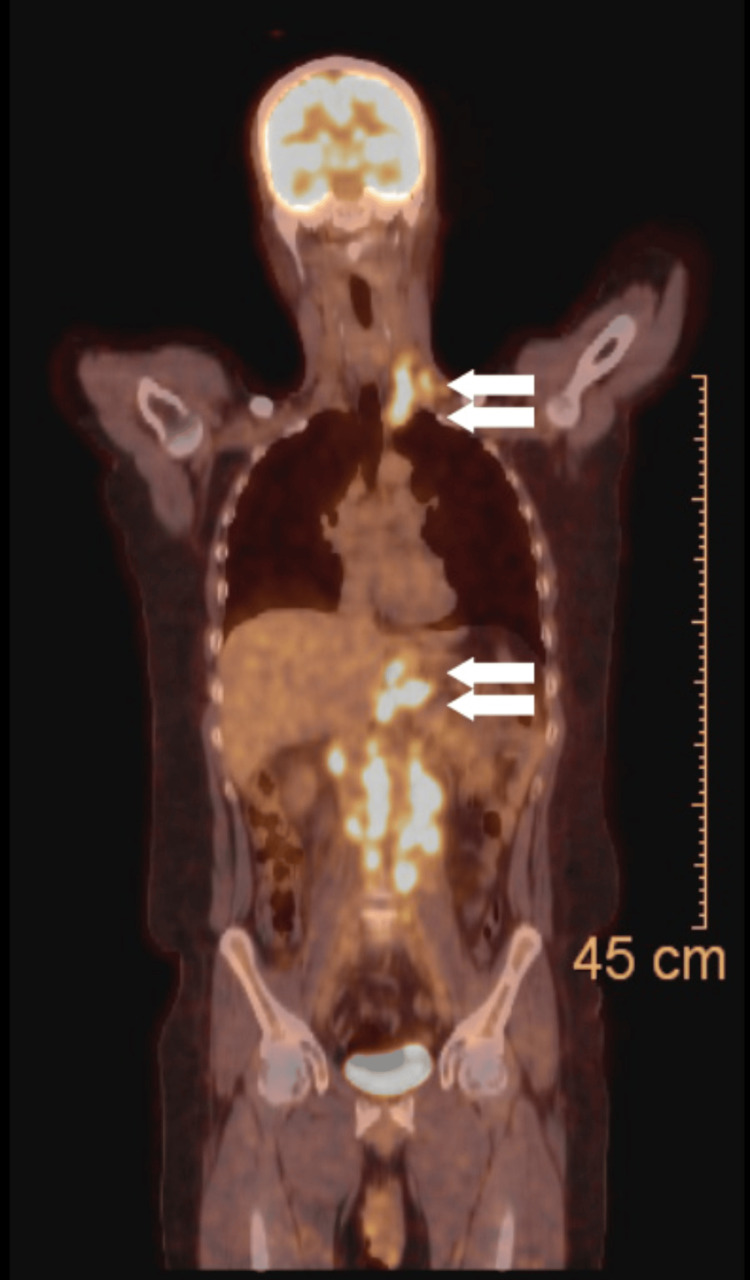
Positron emission tomography-computed tomography scan showing hypermetabolic activity at the gastric fundus extending to the gastroesophageal junction and the distal end of the esophagus and multiple enlarged hypermetabolic metastatic lymph nodes in the left lower neck and mediastinal paraesophageal lymph nodes.

Positron emission tomography-computed tomography scan showed hypermetabolic thickening in the proximal stomach extending to the gastroesophageal junction and the distal end of the esophagus, multiple enlarged hypermetabolic metastatic lymph nodes in the gastrohepatic and retroperitoneal regions, multiple enlarged hypermetabolic metastatic lymph nodes in left lower neck region, and enlarged hypermetabolic mediastinal paraesophageal lymph nodes (Figures 2).

As part of the staging workup and after obtaining consent from the patient, he underwent staging laparoscopy with intraoperative findings of a palpable mass at the gastroesophageal junction, mobile stomach, and multiple enlarged lymph nodes at stations 2 and 7. Fluid cytology was sent and came out to be negative.

Pathology reports of the EGD biopsies showed poorly differentiated adenocarcinoma of the esophageal and gastric masses. Fine-needle aspiration of the left cervical lymph node also showed metastatic poorly differentiated adenocarcinoma.

His case was discussed in the tumor board meeting with multidisciplinary teams, and the decision was made to start palliative chemotherapy.

## Discussion

Obesity and its association with cancer has received much attention. According to the International Agency for Research on Cancer working group, obesity can increase the risk for 14 different types of cancers [3].

High BMI is reported to have a strong association with esophageal adenocarcinoma [4-6] and gastric cardia adenocarcinoma [7]; however, this association is not as strong as for esophageal cancer [5]. This is most likely because reflux mechanisms can also cause esophageal cancer [7].

Obesity is an important risk factor for GERD and its persistence can lead to serious complications (erosive esophagitis, Barrett’s esophagus, and esophageal adenocarcinoma). Patients with erosive esophagitis, Barrett’s esophagus, and esophageal adenocarcinoma were found to have larger intra-abdominal visceral adiposity than controls using CT-measured abdominal fat composition [8]. Alterations in the levels of adipokines, cytokines, and chemokines, as well as insulin/insulin growth factor pathways, can lead to the progression from inflammation to neoplasia, which is considered another mechanism (GERD-independent mechanism) that can lead to the development of Barrett’s esophagus and esophageal adenocarcinoma [9].

This is the second case of gastroesophageal cancer post-sleeve gastrectomy [10] reported from our institution; however, both patients had their surgery outside our hospital with no preoperative endoscopy as both patients did not report GERD symptoms at that time. In our institution, routine preoperative EGD is performed for all patients regardless of the presence of GERD symptoms to look for evidence of esophagitis, *Helicobacter pylori*, hiatal hernia, or any other incidental findings. Postoperatively, we have a strict follow-up program (two years for post-sleeve gastrectomy, following which they can be followed up in primary healthcare centers and lifelong follow-up for the other bariatric procedures) to ensure and monitor the weight reduction of our patients and detect early those with alarming symptoms for any gastroesophageal disease.

Preoperative EGD has been recommended for all patients seeking bariatric surgery due to the large number of incidental findings of gastroesophageal pathologies such as hiatal hernia, esophagitis, gastritis, Barrett’s esophagus, peptic ulcer disease, or upper gastrointestinal tumors [11]. However, currently, the decision to perform preoperative upper gastrointestinal endoscopy is individualized according to the surgeon’s preference, the patient, and the type of surgery [12].

The International Federation for the Surgery of Obesity and Metabolic Disorders 2020 Position Statement reported that 3.8% of obese patients have Barrett’s esophagus and 1.9% of patients will develop Barrett’s esophagus irrespective of the type of surgery [13]. Hence, attention should be paid to patients at high risk of developing malignancy post-bariatric surgery, which include male sex, smoking, severe obesity, a metabolic disease with a high likelihood of centralized obesity, and remarkably high BMI at a young age [14]. Genetic factors (related to the presence of higher inflammatory markers) could be linked to the progression of Barrett’s esophagus to esophageal adenocarcinoma and are still under investigation [14].

## Conclusions

The association between the most important risk factors for developing gastric/esophageal cancer (symptomatic/silent GERD, Barrett’s esophagus) in obese patients should highlight the importance of routine preoperative EGD as an important tool that can be used as a baseline study for patients seeking bariatric surgery to either diagnose asymptomatic esophageal/gastric lesions and esophagitis or to identify and categorize patients according to their findings for future post-operative surveillance plans, especially for patients identified as high risk to develop gastric/esophageal cancer.
